# PD41 Potential Life Lost And Indirect Costs Of Ovarian Cancer For Patients In Brazil: A Real-World Study

**DOI:** 10.1017/S0266462325101748

**Published:** 2025-12-29

**Authors:** Pamela Santana, Eduardo Paulino, Flávia Lopes

## Abstract

**Introduction:**

Ovarian cancer (OC) is the most lethal gynecological tumor worldwide. This study aimed to describe the profile of patients with OC and estimate the indirect costs of OC within the Brazilian Unified Health System (SUS).

**Methods:**

This retrospective observational study used data from the Department of Informatics of the SUS and included patients aged 18 years or older with at least one record of International Classification of Diseases, 10th Revision code C56 from 2014 to 2023. Indirect costs were estimated by calculating years of potential life lost (YPLL) based on a 2022 life expectancy of 79 years for Brazilian women. The age threshold of years of potential productivity lost (YPPLL) was set at 62 years. The cost of productivity loss (CPL) was calculated using the gross domestic product (GDP) per capita in USD from the World Bank, converted to BRL (USD1=BRL5.66).

**Results:**

The study included a total of 36,141 patients with OC, with an average age of 56.1 years (standard deviation 13.4) at the time of their first claim. Most of the patients (51.2%) were white, lived in the southeast region of Brazil (44.6%), and had their first claim at advanced cancer stages (78.1%). The estimated indirect costs for OC were 22.1 YPLL per patient. The estimated YPPLL was 7.3 years, and the CPL was USD65,705.30 (BRL374,040.56) per patient.

**Conclusions:**

This study provided valuable insights into the profile and indirect costs of OC. By analyzing real-world data, we highlighted the burden of OC, which impacts not only the health and longevity of patients but also places a significant financial burden on society due to productivity losses.

